# Pemphigus—A Disease of Desmosome Dysfunction Caused by Multiple Mechanisms

**DOI:** 10.3389/fimmu.2018.00136

**Published:** 2018-02-01

**Authors:** Volker Spindler, Jens Waschke

**Affiliations:** ^1^Department of Biomedicine, University of Basel, Basel, Switzerland; ^2^Faculty of Medicine, Ludwig-Maximilians-Universität (LMU) Munich, Munich, Germany

**Keywords:** pemphigus, autoantibodies, cell adhesion, desmosomes, keratinocytes

## Abstract

Pemphigus is a severe autoimmune-blistering disease of the skin and mucous membranes caused by autoantibodies reducing desmosomal adhesion between epithelial cells. Autoantibodies against the desmosomal cadherins desmogleins (Dsgs) 1 and 3 as well as desmocollin 3 were shown to be pathogenic, whereas the role of other antibodies is unclear. Dsg3 interactions can be directly reduced by specific autoantibodies. Autoantibodies also alter the activity of signaling pathways, some of which regulate cell cohesion under baseline conditions and alter the turnover of desmosomal components. These pathways include Ca^2+^, p38MAPK, PKC, Src, EGFR/Erk, and several others. In this review, we delineate the mechanisms relevant for pemphigus pathogenesis based on the histology and the ultrastructure of patients’ lesions. We then dissect the mechanisms which can explain the ultrastructural hallmarks detectable in pemphigus patient skin. Finally, we reevaluate the concept that the spectrum of mechanisms, which induce desmosome dysfunction upon binding of pemphigus autoantibodies, finally defines the clinical phenotype.

## Introduction

Pemphigus is a severe autoimmune-blistering skin disease caused by autoantibodies primarily targeting the desmosomal adhesion molecules desmogleins (Dsgs) 1 and 3 (1), which are required for the firm intercellular adhesion of keratinocytes. Autoantibodies targeting Dsg3 are found during the mucosal-dominant phase in pemphigus vulgaris (mPV) which is frequently followed by a mucocutaneous phase (mcPV) with additional epidermal blistering and characterized by the presence of both anti-Dsg3 and anti-Dsg1 antibodies. By contrast, in pemphigus foliaceus (PF), flaccid blisters are found in the skin only, and their formation is associated with the occurrence of anti-Dsg1 autoantibodies. Autoantibodies against other desmosomal cadherins such as desmocollin (Dsc) 3 are rarely detectable in PV or PF but can be pathogenic (2–5). The pathogenicity of autoantibodies against a range of non-desmosomal autoantibodies that are often present in patients’ sera in addition to anti-Dsg antibodies is unclear (6). We here focus on the mechanisms causing skin blistering in response to autoantibodies against Dsg1 and Dsg3. In addition to suppressing autoimmunity, which is the current basis for disease management (1), we believe that novel targeted therapeutic strategies are necessary to stabilize desmosomes in situations when autoantibodies are present. It is essential to better understand the regulation of desmosomal adhesion, and we aim to provide perspectives for future research by reevaluating the mechanisms leading to desmosome dysfunction, loss of keratinocyte cohesion, and blistering.

## Histology and Ultrastructure of Patient’s Lesions Highlight Relevant Mechanisms

**Figure 1:**
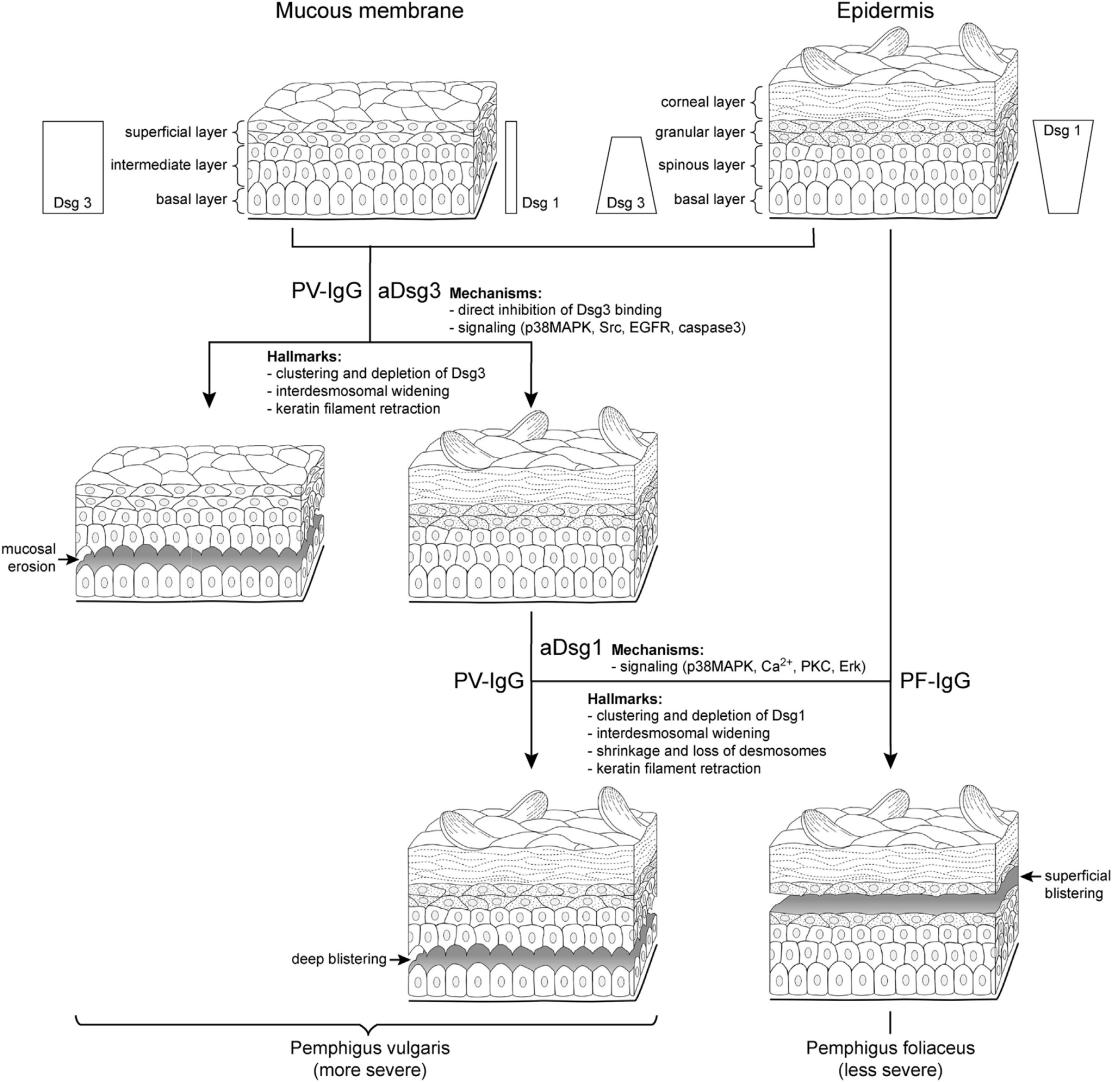
Clinical phenotypes correlate with autoantibody profiles and mechanisms causing desmosome dysfunction. It is well established that clinical phenotypes of pemphigus largely correlate with autoantibody profiles but the underlying mechanisms are not clear. In pemphigus vulgaris (PV) (left), mucosal erosions often precede epidermal blistering. During the mucosal-dominant phase, autoantibodies (PV-IgG) primarily against desmoglein (aDsg3) are pathogenic, which is the most abundant Dsg isoform in the mucosal epithelium. The occurrence of antibodies targeting Dsg1 (aDsg1), which is strongly expressed in the superficial epidermis compared to Dsg3, usually correlates with skin blistering, affecting the deep epidermis right above the basal layer. By contrast, in pemphigus foliaceus (PF), autoantibodies (PF-IgG) primarily against Dsg1 (aDsg1) cause superficial epidermal blistering. The different phenotypes are characterized by structural hallmarks in patients’ lesions, and the mechanisms causing desmosome dysfunction in response to autoantibody binding appear to be different for aDsg1 and aDsg3. Please note that the distribution of Dsg1 and Dsg3 in the epidermis is indicated for all layers except the for corneal layer.

Cultured keratinocytes or mouse models can only partially reproduce the situation in patients. We believe that for the identification of relevant mechanisms in pemphigus pathogenesis, the careful evaluation of patients’ lesions is the gold standard. On the histological level, skin blistering in PV occurs by suprabasal splitting (Figure 1), whereas PF is characterized by superficial lesions restricted to granular or upper spinous layers of the epidermis (1). Although exceptions occur, PF splitting is usually found in the upper half of the epidermis, whereas PV affects the lower half. At least in some parts of typical PV but not PF lesions, the histological hallmark of a “tomb-stone appearance” of keratinocytes in the blister bottom can be detected (7, 8).

By immunostaining, the clustering of Dsg3 together with autoantibodies has been detected in the mucosal and skin lesions in mcPV but also in unaffected epidermis in the mucosal-dominant type (mPV) in the absence of ultrastructural alterations of desmosomes (9, 10). By contrast, Dsg1 clustering was found in mcPV skin and PF epidermis only, i.e., when antibodies against Dsg1 were present (9, 10) (Figure 1). Since Dsg3 clustering was shown to be the structural correlate of Dsg3 depletion (11), these data indicate that the depletion of Dsg3 alone may be a primary mechanism in mucosal eroding but alone is not sufficient to cause epidermal blistering. Nevertheless, Dsg3 depletion may drastically worsen desmosome destabilization in the epidermis which is suggested by the fact that glucocorticoids rapidly improve the clinical phenotype (1) at least in part *via* Stat3-induced Dsg3 transcription increase (12).

On the ultrastructural level, smaller desmosomes were found only in conditions when patients presented with antibodies against Dsg1 such as in mcPV and PF but not in mPV (9, 10, 13, 14), suggesting that Dsg1 targeting is critical and may interfere with desmosome assembly or cause dismantling of existing desmosomes (Figure 1). Besides a reduced size, a general loss of desmosomes is present under all conditions where blistering occurred. Electron microscopy revealed the formation of double-membrane structures in PV and PF containing desmosomes with reduced size and altered morphology which may be the correlate for the depletion of extradesmosomal Dsg molecules and the uptake of entire desmosomes (13). Similarly, interdesmosomal widening, which is the first ultrastructural sign to be detected in pemphigus lesions, may be caused by the endocytosis of extradesmosomal Dsg1 rather than of Dsg3 (13, 15). This alone appears not to be sufficient for blister formation since it was detected also in the unaffected deep epidermis and the mucosa of PF patients but not in mPV with intact Dsg1 distribution.

Split desmosomes both with and without attached keratin filaments were detected by electron microscopy and SIM on the keratinocyte surface facing blisters in PF and mcPV (13, 14). Desmosome splitting can be induced by mechanical stress (14) and may be the ultrastructural correlate for the direct inhibition of Dsg binding. Since split desmosomes in this study were of reduced size, altered desmosome structure appears to be required, suggesting an additional role of impaired desmosome assembly or the depletion of desmosomal Dsg. The final hallmark described early for both PV and PF by electron microscopy is keratin retraction (16, 17) (Figure 1). Recently, keratin filament retraction was observed only when desmosomes were completely absent (13). This can be interpreted in the way that keratin filaments are not the cause but rather the consequence of desmosomal loss or the changes are temporally tightly correlated.

Apoptosis is not a major mechanism because cells displaying signs of apoptotic cell death are absent or sparse in PV and PF skin lesions and therefore cannot explain acantholysis of a significant epidermal area (13, 18, 19).

## Autoantibody-Triggered Mechanisms Impairing Desmosome Turnover

As outlined earlier, split desmosomes, reduced desmosome numbers and size, and keratin retraction are ultrastructural hallmarks in pemphigus skin. Reduced desmosome size or numbers cannot be explained by the direct interference of pemphigus autoantibodies with Dsg binding but rather are a consequence of the altered turnover of desmosomal proteins. These changes are likely steered by intracellular-signaling pathways, which are modulated in response to autoantibody binding and represent potential pharmacologic targets. In principal, reduced desmosome size and numbers can result either from interference with desmosome assembly or from the enhanced disassembly of desmosomes. Available data suggest that in pemphigus, both mechanisms contribute to impaired desmosome turnover, shifting the balance toward an overall reduction of desmosomal components (20).

**Figure 2:**
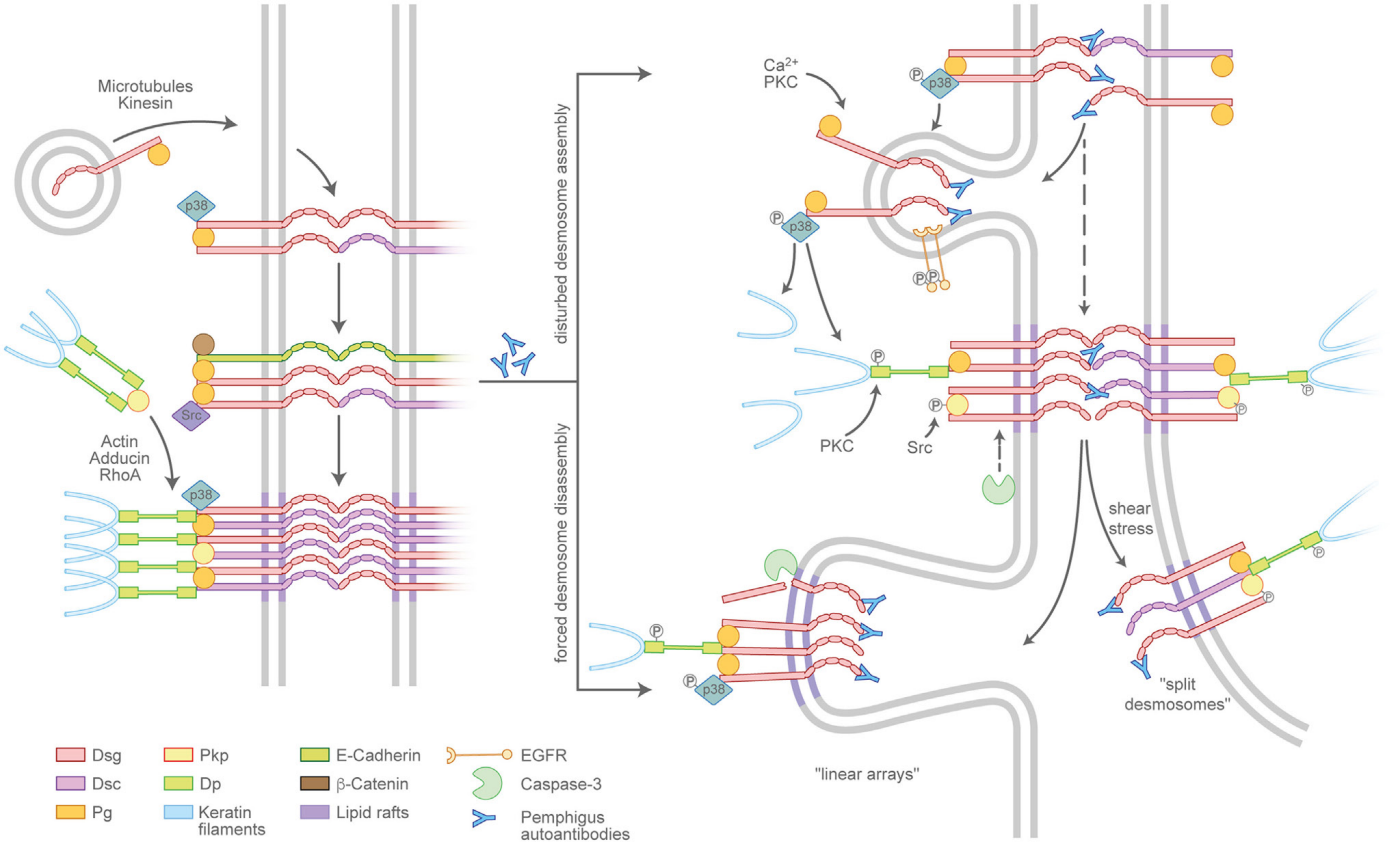
Pemphigus autoantibodies interfere with desmosome turnover. Left panel: a simplified view of the assembly pathway of desmosomes. Desmosomal cadherins and the plaque components desmoplakin (DP), plakophilins (Pkps), as well as the keratins are transported to the plasma membrane by distinct routes and may involve an intermediate complex containing adherens junction proteins. Right panel: Pemphigus autoantibodies interfere with the assembly of nascent desmosomes and promote the disassembly of existing desmosomes. The modulation of desmosome turnover is tied to changes of intracellular-signaling cascades which, for instance, lead to the phosphorylation or cleavage of desmosomal components. Linear arrays represent zones of desmosome disassembly. Split desmosomes may occur if the already-weakened desmosomes are exposed to shear stress.

Desmosome assembly is tightly interwoven with adherens junction formation and appears to proceed in distinct steps (21) (Figure 2, left panel). Desmosomal cadherins are initially transported to the cell membrane in a microtubule- and kinesin-dependent process (22), which, in case of Dsg2, is enhanced by its palmitoylation (23). The precise mechanisms are unclear but once membrane-localized, desmosomal cadherins appear to cluster in an intermediate junction with E-cadherin, β-catenin, and plakoglobin and probably segregate to form desmosomes clusters later on (24, 25). Plakophilins (Pkps) are essential as they are required to assemble keratin-anchored DP pools in the cortical regions of the cell (26, 27). Pkp3 was shown to participate in transferring DP clusters to the membrane and to stabilize desmosomal cadherins in a Rap1-dependent manner (28). In addition, cortical actin and actin-binding proteins such as adducins and RhoA signaling are necessary for full desmosome assembly (29–31). Desmosomal molecules localize to lipid rafts and the raft-associated proteins Flotillin-1 and -2 (32, 33). In line with this, interference with lipid raft composition prevents both desmosomal assembly and disassembly, suggesting these lipid-enriched membrane domains to be hot spots for desmosome turnover. Compared to the assembly, the disassembly of desmosomes under physiologic conditions is poorly understood, which may be related to the relative chemical inaccessibility and the stability of maturated desmosomes (34).

Pemphigus autoantibodies interfere with desmosome turnover by enhancing the internalization of desmosomal components (Figure 2, right panel). However, a clear separation of mechanisms specifically affecting either the assembly or the disassembly pathway in most cases is not possible. It is conceivable that autoantibodies reach Dsgs located in extradesmosomal membrane pools more easily than those densely packed in a mature desmosome. In line with this scenario, the non-desmosomal pool of Dsg3 is the first to be depleted (35, 36). Consequently, the reduced availability of supply molecules leads to the destabilization of desmosomes and in the longer run may favor the depletion of desmosome-localized, now more easily accessible molecules. Thus, the reduced desmosome assembly route in pemphigus is at least in part a result of internalized extradesmosomal molecules, summarized as “desmoglein nonassembly depletion hypothesis” (9, 37). However, the situation apparently is more complex than a simple disbalance in supply and demand. Importantly, especially the extradesmosomal molecules are considered to serve as signaling scaffolds (38, 39). As an example, it was shown that a complex of Dsg3 and Pg binds p38MAPK and suppresses its activity (40, 41). Upon the loss of Dsg3 interaction, e.g., by steric hindrance through pemphigus autoantibodies, this suppressive function is abolished and p38MAPK is activated. Indeed, p38MAPK signaling was shown to promote Dsg3 internalization (42) as well as keratin retraction (41, 43), suggesting that p38MAPK is a central signaling molecule in desmosome turnover. *Vice versa*, keratin filaments were shown to influence both desmosome stability and Dsg3-binding characteristics in a signaling-dependent manner. Under physiologic conditions, the keratin-dependent suppression of p38MAPK signaling increases Dsg3-binding strength, whereas the suppression of PKC signaling through the adapter protein Rack-1 prevents DP phosphorylation, stabilizes Dsg3 in the desmosome (44, 45), and, together with Pkp1 (46), promotes a hyperadhesive state. These functions appear to be disturbed by autoantibody-induced keratin retraction (Vielmuth et al., Frontiers Immunol, this issue). Furthermore, Pkp3 is phosphorylated in response to autoantibody binding in an Src-dependent manner (47), which may be connected to Src being present in the intermediate E-cadherin/Dsg3 complex (25). Together, these data suggest a feed-forward loop from extradesmosomal complexes to desmosomes that, at least in part through interference with keratin-dependent signaling, destabilizes desmosome composition and function. As the precise mechanisms are unclear, it is possible that keratins affect both the assembly and the disassembly pathways of desmosomes.

The occurrence of desmosome disassembly can be concluded from observations that Dsg3 together with PV-IgG is excluded from desmosomes and internalized (48). This is supported by observations that IgG autoantibodies can access Dsgs in native desmosomes (49). Clusters of desmosomal molecules localizing in arrays perpendicular to the cell–cell border may represent sites of internalization of single desmosomal components or the entire complexes (50). These may correspond to the “double-membrane structures” that are visible in patients’ skin and are thought to be regions of internalization for partially dismantled desmosomes (13). Although all desmosomal proteins eventually are internalized, the degradation pathways of specific components are not uniform. Dsg3 as well as Pg colocalize with markers for endosomes and lysosomes, whereas DP and presumably other plaque proteins use different, yet unknown, routes (35, 42). Lipid rafts are thought to be important for desmosome disassembly, as the impairment of raft composition prevents the depletion at least of Dsg3 and reduces loss of cell cohesion (32) (Schlögl et al., this issue). In addition, the cleavage of Dsg3 by caspase-3 independent from apoptosis was observed and may promote internalization (51–53), the latter of which was shown to require EGFR signaling (54).

Split desmosomes are rarely detectable in keratinocyte cultures incubated with pemphigus antibodies in the absence of mechanical stress (55) but are enhanced when cultures are subjected to mechanical strain as well as in human skin models and in patients’ skin (14, 56). Furthermore, split desmosomes appear typically severely altered with reduced plaque sizes and aberrant keratin insertion. This may indicate that desmosome splitting occurs secondary to changes in desmosome composition. Collectively, interference with desmosome turnover can largely explain the ultrastructural hallmarks of pemphigus patients’ lesions.

## Desmosome Dysfunction Defines the Clinical Phenotype of Pemphigus

All mechanisms described earlier finally lead to desmosome dysfunction, which impair keratinocyte cohesion. However, the functional interplay of the different mechanisms is not well understood at present. It is also unclear why epidermal splitting affects the deep epidermis in PV, whereas PF blisters are restricted to superficial epidermal layers (Figure 1). Besides homophilic adhesion, Dsgs were shown to undergo heterophilic interactions both with other Dsg isoforms and Dscs, respectively (5, 45, 57, 58). Moreover, the genetic deletion of Dsc3 causes a PV phenotype with suprabasal blistering in mice (59). On the other hand, the forced overexpression of Dsg2, a Dsg isoform not present in the adult human epidermis except hair follicles, which is upregulated in PV lesions, can compensate for the loss of Dsg1 in a PF mouse model (60, 61). Therefore, it is likely that all desmosomal cadherins (i.e., Dsg1–4, Dsc1–3) contribute to keratinocyte cohesion and epidermal integrity in a layer-specific manner.

In the mucosa, Dsg3 is the predominant desmosomal cadherin, and in the superficial epidermis Dsg1 is strongly expressed, whereas other isoforms of desmosomal cadherins are largely absent except of Dsc1 (Figure 1) (8). The cadherin distribution pattern can well explain the clinical phenotype of mPV and PF because the loss of function of the target molecules cannot be outbalanced by relevant amounts of other desmosomal cadherins (1). However, the Dsg compensation theory falls short in mcPV, because if Dsg1 and Dsg3 would specifically compensate for each other, the whole epidermis or at least the lower epidermis should disintegrate completely when considering that autoantibodies entering the epidermis from the dermis may be concentrated most in the first few layers of cells. Rather, epidermal splitting typically occurs right at the suprabasal level, i.e., between the basal keratinocytes and the first layer of the spinous layer cells where in intact epidermis as well as in PV lesions, Dsg1 and Dsg3 are expressed (8). This favors the hypothesis that different mechanisms contribute to desmosome dysfunction in a layer-specific manner.

The direct inhibition of Dsg3 binding by PV-IgG has been described under cell-free conditions as well as on the surface of living keratinocytes; however, it was ineffective to induce the complete loss of cell cohesion (62–64). By contrast, the interference of anti-Dsg1 autoantibodies with Dsg1 binding was detectable neither in cell-free AFM experiments (63, 65, 66) nor in studies with intact keratinocytes (Vielmuth 2018, Frontiers Immun, this issue). This suggests that the mechanisms underlying the loss of keratinocyte cohesion in pemphigus are autoantibody-specific. In this respect, it was reported recently that autoantibody profiles in pemphigus correlate with signaling patterns which makes it intriguing to speculate that specific signaling patterns may be defining the clinical phenotype (65). It has been reported that p38MAPK and Src can be activated by PV-IgG containing antibodies against Dsg3 as well as by AK23 which is Dsg3-specific (Figure 1). In addition, EGFR and caspase-3 activation in response to AK23 and PV-IgG was shown in mouse models (53, 54, 67). By contrast, the increase of intracellular Ca^2+^ as well as Erk activation was induced only by PV-IgG and PF-IgG containing Dsg1 autoantibodies, which was paralleled by p38MAPK activation (65). In this line of thoughts, the direct inhibition of Dsg3 binding together with the signaling of p38MAPK, Src, EGFR, and caspase-3 would be sufficient to cause mucosal erosions (Figure 1), whereas additional mechanisms such as Ca^2+^, which has been shown to be associated with PKC activation in keratinocytes treated with pemphigus antibodies (68, 69), as well as Erk would be required for epidermal blistering. If so, it could be postulated that one function of Dsg and Dsc isoforms expressed in the epidermis but not in the mucosa may be to further strengthen keratinocyte cohesion by regulating signaling pathway which control desmosome turnover and function.

For most of the pathways described earlier, it was shown that modulation is sufficient to largely reduce skin blistering in mice (6). However, mice differ from humans with respect to epidermal Dsg expression patterns. Moreover, the genetic deletion of Dsg3 or the inactivation of Dsg3 *via* incubation with AK23 is sufficient to cause skin blisters in mice (41, 70). This is different to the situation in mPV where the skin is usually not affected and indicates that Dsg3 is more critical for epidermal integrity in mice compared to humans. Therefore, to prevent misinterpretations, studies including ultrastructural analyses of human skin are required in addition to mouse models. Indeed, studies in human skin *ex vivo* indicate that the significance of p38MAPK for blister formation may be different in PV and PF. When using PV-IgG, the inhibition of p38MAPK was sufficient to abrogate blistering (11, 56) but not when PF-IgG was applied (71). On the other hand, it was shown that p38MAPK in response to PV-IgG and PF-IgG is involved in the reduction of desmosome length (56, 71). However, since the shortening of desmosomes and interdesmosomal widening were detectable also in the absence of blistering following treatment with AK23, this indicates that p38MAPK-mediated reduction of desmosome length and interdesmosomal widening may not be sufficient to cause skin blistering. Rather, the complete loss of desmosomes appears to be required (56) for which other mechanisms such as Ca^2+^, PKC, or Erk may be necessary to induce superficial epidermal blistering in PF (Figure 1). However, when bolstered by the direct inhibition of Dsg3 binding and Src activation, the panel of mechanisms causing desmosome dysfunction may be sufficient to cause suprabasal splitting as seen in mcPV. Taken together, it is likely that the clinical phenotype in pemphigus is caused by a complex set of mechanisms causing desmosome dysfunction.

## Author Contributions

VS and JW conceived the perspective and wrote the manuscript.

## Conflict of Interest Statement

The authors declare that the research was conducted in the absence of any commercial or financial relationships that could be construed as a potential conflict of interest.
